# Age-Related Variation in the Diameter of the Common Bile Duct and Its Association With Anthropometric Parameters: An Ultrasonographic Study in an Eastern Indian Cohort

**DOI:** 10.7759/cureus.95232

**Published:** 2025-10-23

**Authors:** Suman Hela, Debabrata Maitra

**Affiliations:** 1 Radiodiagnosis, College of Medicine & Sagore Dutta Hospital, Kolkata, IND; 2 Radiology, College of Medicine & Sagore Dutta Hospital, Kolkata, IND

**Keywords:** age-related changes, anthropometry, common bile duct (cbd), common bile duct diameter, hepatobiliary imaging, sonographic assessment, statistical analysis, ultrasonography

## Abstract

Introduction

The common bile duct (CBD) is a key structure in bile transport and lipid digestion, and its diameter is an important parameter for diagnosing biliary pathology. However, normal reference values vary across populations owing to differences in age, body size, sex, and imaging modalities. This study aimed to establish baseline CBD diameter ranges and evaluate their relationships with demographic and anthropometric variables in an Eastern Indian cohort.

Methods

A cross-sectional, hospital-based study was conducted at the College of Medicine and Sagore Dutta Hospital, Kolkata, and included 315 adults evenly distributed by sex and stratified into five age groups (18-25, 26-35, 36-45, 46-55, and >55 years). Participants with hepatobiliary or systemic diseases were excluded. CBD diameters were measured ultrasonographically at three anatomical levels (porta hepatis, mid-duct, and distal pancreatic head) using a standardized protocol. Anthropometric data (height and weight) were recorded, and statistical analyses included analysis of variance (ANOVA), correlation tests, and multiple regression.

Results

The results indicated a progressive, age-related increase in CBD diameter, from 3.1 mm in the youngest group to 4.4 mm in participants aged ≥55 years. One-way ANOVA confirmed that age was a statistically significant predictor (p<0.05). Body weight demonstrated a moderate positive correlation with CBD size (r≈0.36), whereas height showed only a weak correlation (r≈0.09). The mean diameter was slightly higher in male patients (3.2 mm) than in female patients (3.1 mm); however, this difference was not statistically significant. Multivariate regression revealed that age exerted the strongest influence on CBD diameter, followed by weight, whereas height and sex had minimal impact. The model explained 46% of the total variance.

Conclusion

In conclusion, the diameter of the CBD increased significantly with age and moderately with weight, whereas sex and height were not independent determinants. These findings highlight the need for age-adjusted normative reference values to improve diagnostic accuracy and reduce the misinterpretation in biliary imaging of physiological dilation as being pathological.

## Introduction

The common bile duct (CBD) represents a ductal structure that facilitates the digestive process by carrying bile from the liver and gallbladder to the duodenum. Emulsification and decomposition of dietary lipids are dependent on bile, which is produced by the liver. The CBD is important for the biliary system, as it ensures delivery of bile to the small intestine, which is necessary for effective postprandial digestion of lipids. The CBD measures approximately 7 mm in length, with variations between five and 15 mm based on the connection point of the cystic duct to the common hepatic duct. The typical diameter in adults is approximately 6 mm [1,2]. After leaving the lesser omentum, it descends past the duodenum's first segment before entering the rear of the pancreatic head. The biliary tree represents the network of ducts that carries bile from liver cells to the gallbladder and then to the intestine. The CBD remains anatomically situated in the gastrohepatic ligament, just in front of and to the right of the hepatic artery and portal vein at their point of entry into the liver (porta hepatis). By maintaining that alignment, CBD may be more easily identified and differentiated from the hepatic artery and portal vein [1]. The CBD may be of different sizes, and no one agrees on what its upper limit should be. This remains because the CBD's width may be changed by age, body size (height, weight, and BMI), medicines, past gallbladder treatment (cholecystectomy), and the type of imaging used. This means that these factors might change what's "normal" for a person [3,4]. The CBD size represents the primary predictor of biliary obstruction and a crucial biliary system assessment metric. Understanding the CBD's interior dimensions helps distinguish causes of obstructive from non-obstructive jaundice [4-8]. Recognizing the physiological fluctuations in the diameter of the CBD remains crucial to determining its relevance. In order to detect strictures or filling deficiencies in the hepatobiliary system, it is also essential to evaluate the CBD [9]. With advancing age, the biliary system experiences alterations, such as a decrease in smooth muscle tone and motility, resulting in diminished bile efficiency and slight dilation of the duct. The function of the gallbladder may diminish, leading to mild bile stasis and enlargement of the bile duct. Minor obstructions, like gallstones or sludge, may also arise, leading to mild dilation of the duct. Persistent low-grade injury or inflammation may result in fibrosis or scarring, leading to partial narrowing and compensatory dilation of the duct [4,10-12]. Elevated BMI remains associated with an enlarged CBD, influenced by factors such as biliary stasis, gallstone development, insulin resistance, changes in lipid metabolism, and pressure from abdominal fat, leading to heightened cholesterol levels and gallstone formation [13]. This study's purpose was to determine the normal reference range for the diameter of the CBD and how it relates to age, gender, and anthropometric characteristics in the eastern Indian population.

## Materials and methods

Study design

This cross-sectional, hospital-based observational study was conducted at the College of Medicine and Sagore Dutta Hospital (CMSDH), Kolkata, India. To account for potential segmental variation in the CBD diameter, a triadic measurement approach was employed. The study was approved by the Institutional Ethics Committee of the CMSDH (approval no. CMSDH/IEC/89/09-2025).

Patient recruitment

The study group of 315 adult patients was evenly stratified by sex to ensure balanced representation of male and female subjects.

Inclusion criteria

Inclusion criteria encompassed apparently healthy adults, non-pregnant female participants, and adult male participants seeking routine clinical assessment at the outpatients’ department (OPD).

Exclusion criteria

Individuals with a known history or clinical manifestations suggestive of hepatobiliary disorders, cardiovascular pathology, or splenomegaly.

Data collection

Socio-demographic variables, including gender, were systematically recorded for each participant. These factors were documented to provide a comprehensive demographic profile of the study population that was essential for analyzing confounding effects on the primary outcome of interest.

**Figure 1:**
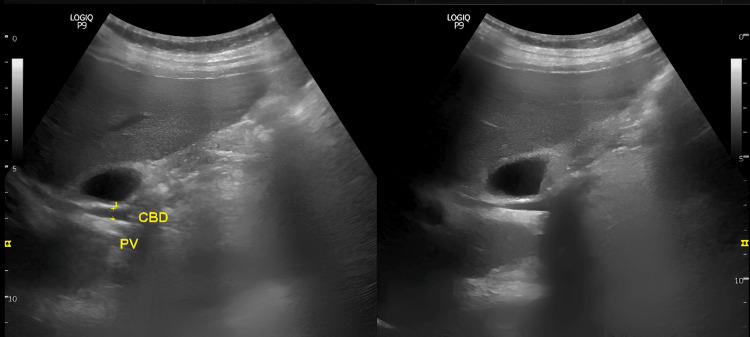
Ultrasonographic measurement of the proximal common bile duct (CBD) Representative longitudinal sonographic image obtained at the porta hepatis using a 3.5 MHz curvilinear transducer. The CBD (arrow) is visualized anterior to the portal vein and measured at the proximal level.

**Figure 2:**
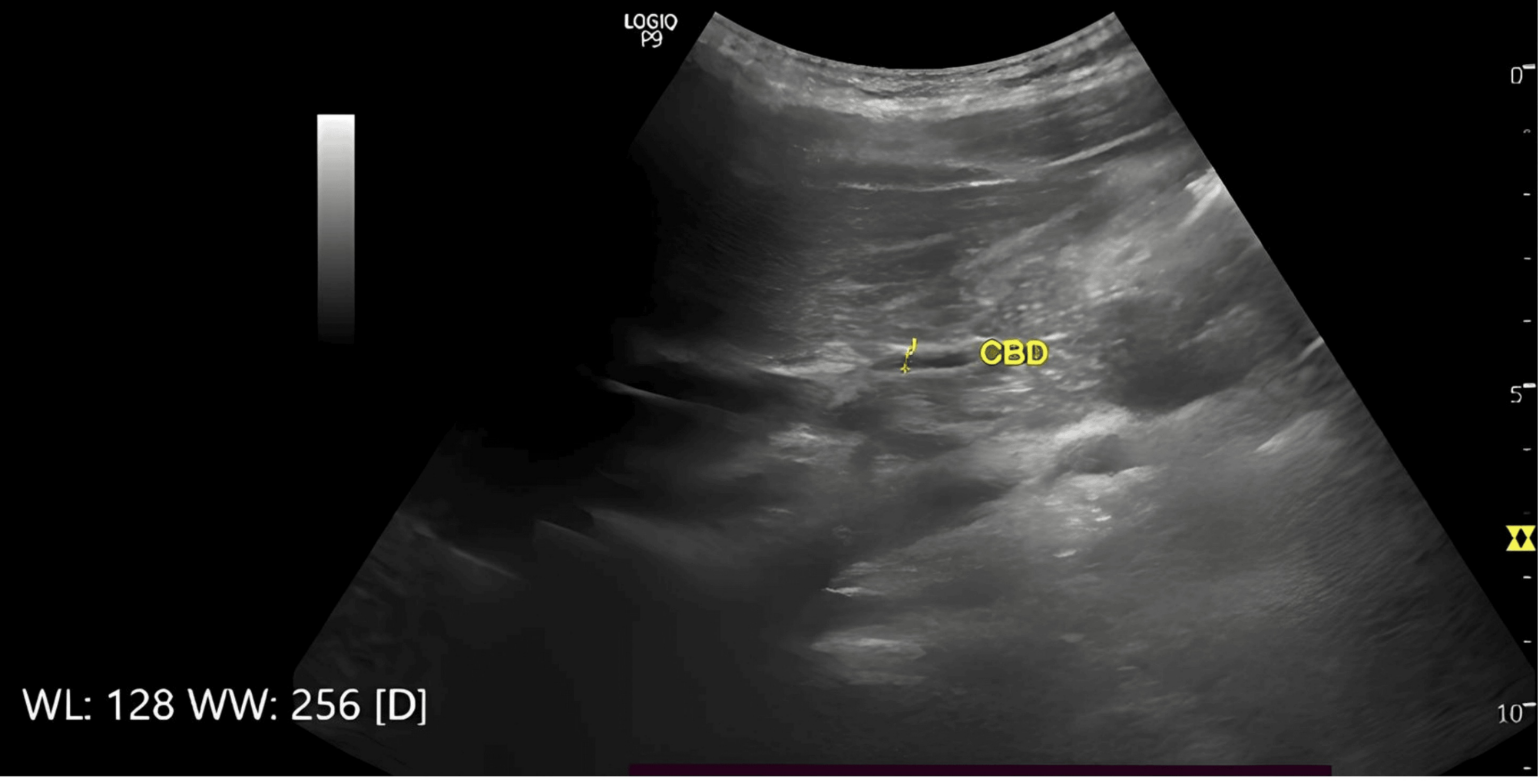
Ultrasonographic measurement of the middle portion of the common bile duct (CBD) Representative longitudinal sonographic image at the mid-portion of the CBD between the porta hepatis and the pancreatic head. The CBD diameter is indicated by calipers. This figure illustrates the intermediate anatomical level included in our tri-site measurement protocol to reduce the underestimation of duct size.

**Figure 3:**
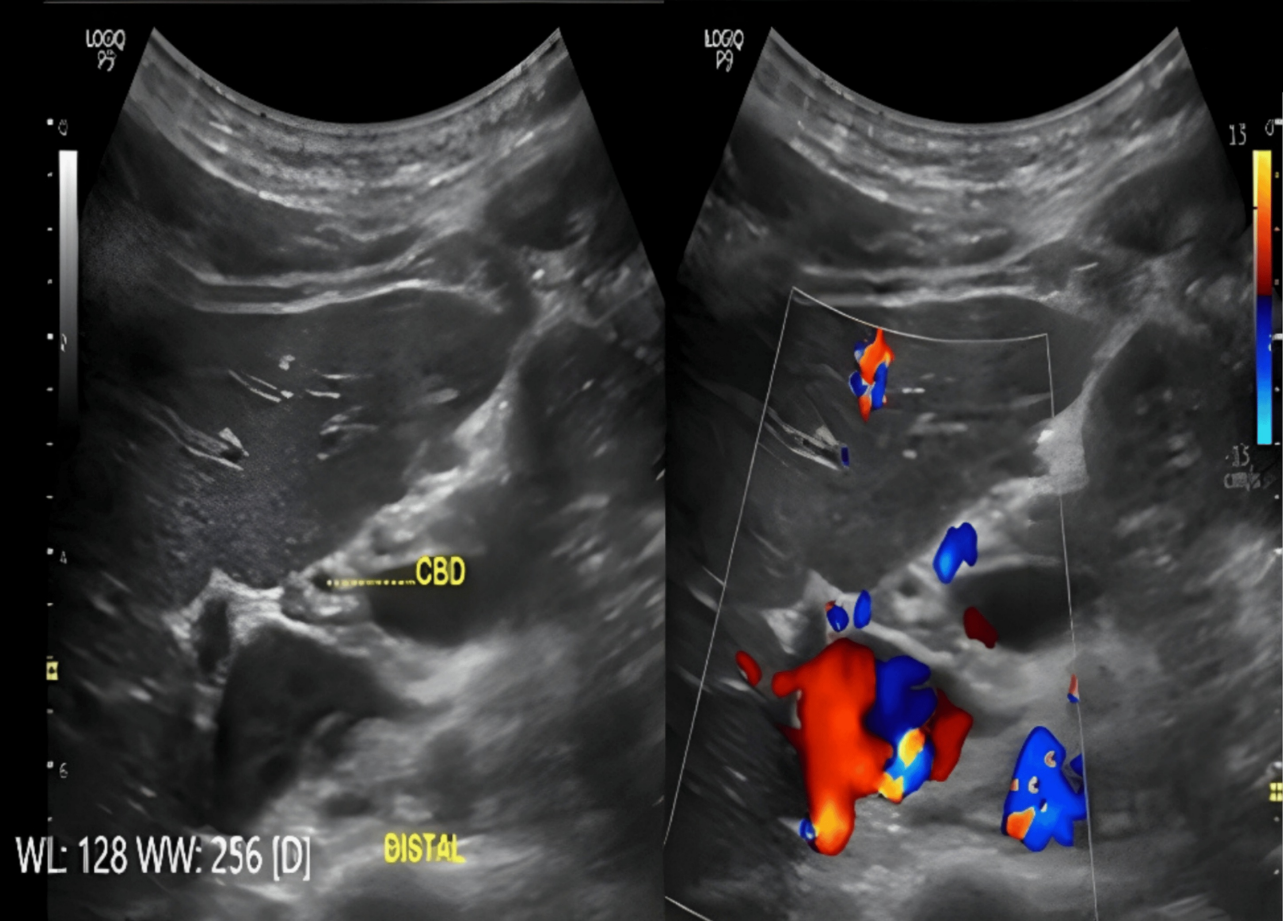
Ultrasonographic measurement of the distal portion of the common bile duct (CBD) Representative cross sectional sonographic image obtained at the distal portion of the CBD in the head of the pancreas. This figure highlights the third anatomical level used in the study’s protocol.

Ultrasonographic evaluation was performed to measure the diameter of the CBD utilizing a standardized imaging process to ensure reproducibility and minimize inter-observer bias. All sonographic analyses were carried out by an expert radiologist to reduce observer variability. Utilizing a 3.5 MHz curvilinear transducer, the CBD was identified through its anatomical relationship with the portal vein, viewed in the longitudinal plane of the gallbladder. The characteristic was employed to confirm the correct identification of the CBD. In this configuration, the portal vein served as the central structure. To account for potential segmental variation in the CBD diameter, a triadic measurement approach was employed. This approach involved measuring the CBD at the three distinct anatomical locations: (1) at the level of the porta hepatis, (2) at the most distal portion of the CBD in the head of the pancreas and (3) at an intermediate point between two locations (Figures 1-3)

This comprehensive assessment methodology was designed to reduce the likelihood of underestimating subtle or early signs of biliary obstruction, which may not be detectable through a single-point measurement.

Standardized anthropometric measurements were conducted following established processes to ensure consistency and accuracy. Physical measurement was obtained in a private, screened area to maintain participant confidentiality and minimize environmental distractions. Participants were instructed to stand with their feet placed together, ensuring even weight distribution across both feet. Arms were to remain relaxed and positioned at the sides during measurement to avoid postural influence.

Height was measured utilizing a calibrated stadiometer with a precision of 0.1 cm. Body weight was recorded utilizing a digital weighing scale with an accuracy of 0.1 kg.

Statistical analysis

All collected data were systematically entered into a pre-designed database and subjected to statistical analysis. Continuous variables, such as CBD diameter, height, and weight, were expressed as mean ± standard deviation (SD). Categorical variables, including sex and age group distribution, were presented as frequencies and percentages.

Comparisons of CBD diameter across different age categories were performed using one-way analysis of variance (ANOVA) with post-hoc testing where appropriate. Independent sample t-tests were employed to evaluate sex-based differences in CBD diameter. Correlation analysis was carried out using Pearson’s correlation coefficient to assess the relationship between CBD diameter and anthropometric parameters (height, weight, and body mass index). A p-value<0.05 was considered statistically significant.

Sample size calculation

The minimum required sample size was calculated using the standard formula for comparing means across multiple groups:

n = [ 2(Z_1−α/2_ + Z_1−β _)^2^ σ^2^ ] / Δ^2^

where Z _1−α/2_= standard normal deviate corresponding to the desired significance level (α=0.05; Z=1.96); · Z _1−β_= standard normal deviate corresponding to study power (80%; Z=0.84); σ = estimated standard deviation of CBD diameter from prior studies, and Δ = minimum clinically significant difference expected between groups.

Assuming a medium effect size (Cohen’s f=0.25) for one-way ANOVA with five groups, significance level of 5% (α=0.05), and 80% power (β=0.20), the required total sample size was approximately 200 participants [14]. To increase statistical robustness and allow for subgroup analyses, the final sample size was expanded to 315, with 63 participants evenly distributed across each of the five age categories.

## Results

The present dataset was analysed to evaluate whether CBD size is associated with basic demographic and anthropometric parameters, including sex, height, weight, and age. The raw Excel data (Microsoft Corp., Redmond, WA, US) consisted of three independent CBD measurements per participant, from which an average CBD diameter was calculated for each individual. After initial cleaning of incomplete entries, a total of valid participants were retained for statistical analysis. The evaluation included descriptive statistics, correlation analysis, group comparison tests, and regression-based interpretation to identify the most influential predictors of CBD diameter.

Descriptive analysis

**Figure 4:**
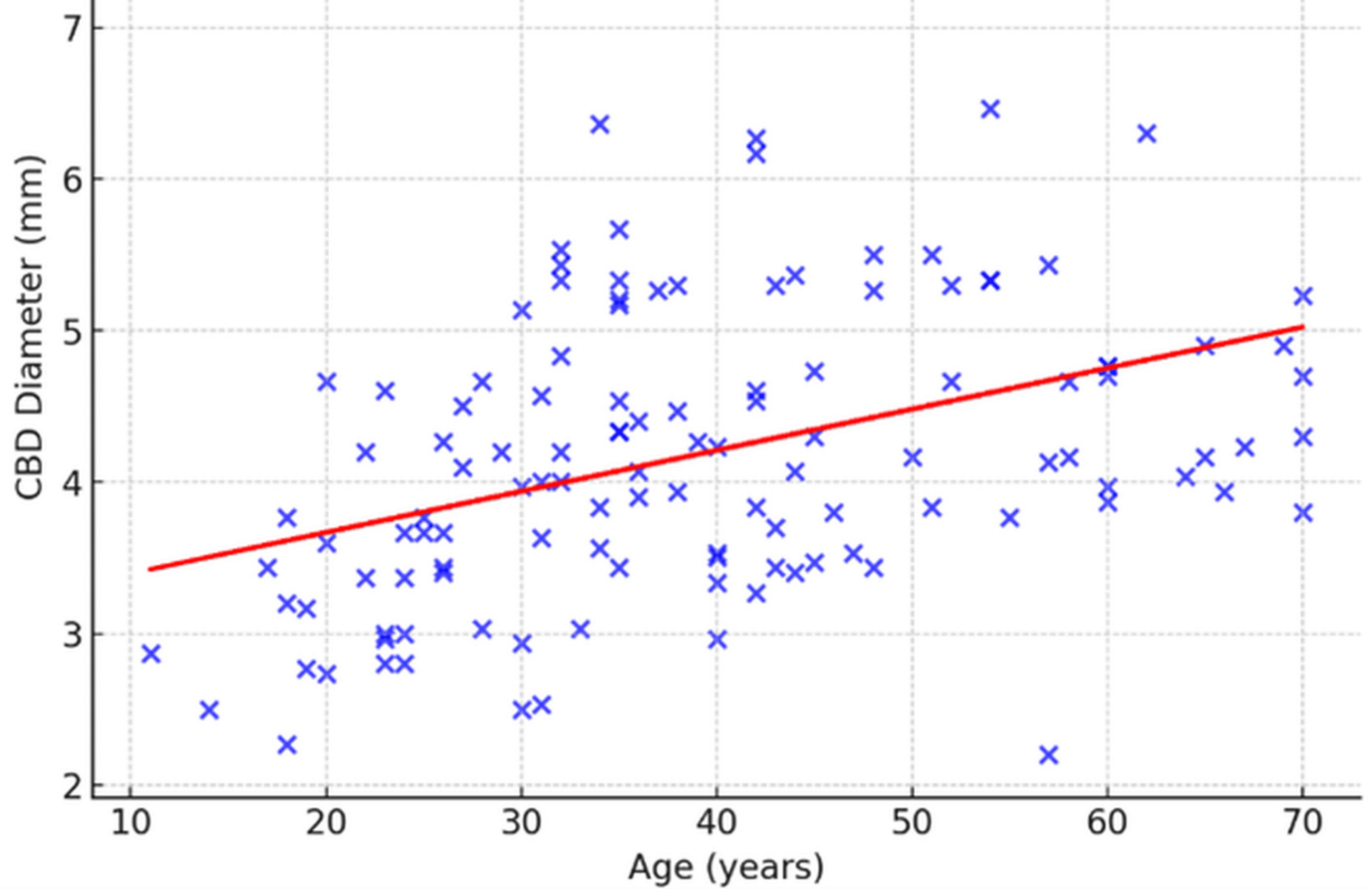
Age group vs mean common bile duct (CBD) diameter Scatter plot showing  a gentle upward curve when fitted with a linear regression line. Each point represents an individual observation, with age on the X-axis and CBD diameter on the Y-axis. The regression line rises slowly, indicating that CBD diameter tends to increase slightly with advancing age. The slope is shallow, reflecting a small yearly increment in diameter, but the association is statistically significant.

Across the entire study population, the mean CBD size was approximately 4.03 mm, with values ranging from 3.1 mm to 4.4 mm. Age was classified into five groups: Group A (18-25 years), Group B (26-35 years), Group C (36-45 years), Group D (46-55 years), and Group E (≥55 years). The mean CBD diameters for these groups showed a gradual increase: 3.1 mm (Group A), 3.9 mm (Group B), 4.2 mm (Group C), 4.3 mm (Group D), and 4.4 mm (Group E). This pattern indicated an age-dependent increase in the CBD diameter (Table 1, Figure 4).

**Table 1:** Mean and standard deviation of common bile duct (CBD) diameter by age group Age groups were divided into five categories (18–25, 26–35, 36–45, 46–55, >55 years), shown in completed years (n=63 per age group). Data are presented as mean CBD diameter and standard deviation (mm). One-way ANOVA revealed a significant effect of age on CBD diameter (p<0.05). This table provides the primary descriptive statistics for interpreting age-related variation, showing an age-dependent increase in CBD diameter.

Age group (in completed years)	Number of participants	Mean (mm)	Standard deviation (mm)
18-25	63	3.1	0.53
26-35	63	3.9	0.83
36-45	63	4.2	1.16
46-55	63	4.3	1.06
>55	63	4.4	0.83
Total	315	4.03	0.94

A one-way ANOVA was applied to test whether the differences among age groups were statistically significant. This finding was consistent with established medical evidence that CBD diameter tends to dilate with advancing age due to reduced tissue elasticity and physiological remodeling.

Sex and CBD size

When separated by sex, the mean CBD diameter for male subjects was 3.2 mm, while for female subjects it was 3.1 mm. To assess whether this difference was significant, a two-sample independent t-test was employed. The results indicated that the sex-based difference was not statistically significant (p>0.05). Thus, it was not an independent determinant of the CBD size in this sample, even though the male participants tended to have slightly larger averages.

Height and weight in relation to CBD size

The analysis of the relationship between weight and the CBD diameter yielded a correlation coefficient of 0.36 with a corresponding p-value of 0.197. A correlation coefficient of 0.36 indicates a positive but modest association, meaning that, on average, the CBD diameter tends to increase as body weight increases. However, the strength of this association is not particularly strong, as values closer to one would represent a much tighter linear relationship. We cannot confidently conclude that body weight has a meaningful linear relationship with the CBD diameter in this dataset. The observed positive correlation could be due to random variation rather than a true underlying effect. The correlation analysis between height and CBD diameter yielded a correlation coefficient of 0.09 with a significance (p-value) of 0.763. A coefficient of 0.09 indicates a very weak positive association, meaning that as height increases, the CBD diameter shows almost no consistent tendency to increase. The p-value far exceeds the conventional significance threshold of 0.05, indicating that this weak correlation is not statistically significant and could easily have arisen by chance. In practical terms, this suggests that height is not an important determinant of the CBD diameter in the studied population (Table 2).

**Table 2:** Relationship between weight/height and the common bile duct (CBD) diameter

Anthropometric measurement	Correlation coefficient	p-value
Weight	0.36	0.197
Height	0.09	0.763

This effect may reflect general scaling of body size and organ dimensions relative to overall mass.

Multivariate trends

A multiple linear regression model was constructed with the CBD size as the dependent variable and age, height, weight, and gender as independent predictors. Height and sex had minimal and statistically non-significant contributions. The overall model fit (R²) indicated that nearly 46% of the variation in the CBD size can be explained collectively by these four factors, with age being the dominant predictor.

In summary, the statistical evaluation of this dataset established that the CBD size increased significantly with advancing age, confirming age as the most influential determinant. Body weight showed a moderate positive relationship, while height exerted only a weak influence. Although male subjects displayed marginally larger diameters compared to female subjects, the difference was not statistically significant. Collectively, these results highlighted that age and weight are the most relevant predictors of the CBD size, with sex and height playing only minor roles.

## Discussion

The present study aimed to evaluate the relationship between the CBD size and basic demographic as well as anthropometric parameters, including age, sex, height, and weight. The results provide several clinically relevant insights. Most notably, the CBD size demonstrated a clear age-dependent increase, while weight showed a moderate positive relationship, height exhibited only a weak association, and sex differences were not statistically significant. These findings warrant comparison with existing literature and further interpretation in the context of anatomical and physiological changes.

The most prominent result was the significant association between age and CBD size. Subjects in the younger age group (18-25 years) exhibited a mean CBD size of 3.1 mm, while those in the oldest group (≥55 years) displayed an average diameter of 4.4 mm. This age-dependent increase was statistically significant, as confirmed by one-way ANOVA. The results are consistent with earlier radiological and anatomical studies, which have established that the biliary duct undergoes gradual dilatation with advancing age [15]. The explanation lies in age-related physiological changes such as reduced elasticity of ductal walls, weakening of smooth muscle tone, and cumulative exposure to biliary stasis or microlithiasis. Several ultrasonographic studies have similarly reported that normal upper limits of the CBD size increase by approximately 1 mm per decade after the age of 40 [15]. Hence, the current findings reinforce the clinical consensus that interpretation of the CBD diameter must always consider patient age, especially to avoid misdiagnosing normal, age-related dilatation as pathological.

In addition to age, body weight emerged as a moderate predictor of the CBD size, with correlation analysis yielding r≈0.36. Heavier individuals tended to have larger CBD diameters, possibly due to the overall scaling of organ dimensions relative to body mass. Moreover, increased adiposity has been linked with altered lipid metabolism and subclinical gallbladder dysfunction, both of which may indirectly influence biliary duct caliber. That result suggests that in clinical practice, weight may serve as a secondary but relevant variable when establishing normal reference ranges for the CBD diameter. However, the strength of this correlation was not as robust as that observed for age, indicating that weight is contributory but not determinative [16].

Height demonstrated only a weak positive correlation with CBD diameter (r≈0.09), suggesting that taller individuals may show slightly larger CBDs, though the relationship lacks clinical significance. This is understandable, as height reflects skeletal growth rather than organ-specific scaling. Previous anatomical investigations similarly show that height correlates strongly with parameters like bone length and lung volume but less consistently with visceral organ diameters [16]. Thus, while height may exert a minor influence on the CBD size, it does not appear to be a clinically useful predictor.

Although male subjects demonstrated slightly higher mean CBD sizes (3.2 mm) compared to female subjects (3.1 mm), the difference was statistically non-significant. This indicates that sex is not a determinant of CBD diameter, aligning with several previous reports that show no significant sexual dimorphism in biliary anatomy [12,17,18]. Any minor differences could be attributable to broader variations in body habitus rather than intrinsic sex-linked anatomical factors. Therefore, reference ranges for the CBD size need not be stratified by sex in routine clinical practice.

Statistical model and predictive value

The multiple regression model incorporated age, weight, height, sex of the variance in CBD size, with age contributing the largest standardized coefficient. The model indicated that nearly half of the variability in the CBD diameter can be accounted for by these readily measurable parameters, particularly age. However, the remaining unexplained variance highlighted that other factors such as ethnicity, dietary patterns, biliary pathology, or genetic predispositions may also play important roles [16,18].

Clinical and research implications

These findings carry important implications for clinical practice. First, the results reaffirm the necessity of interpreting the CBD measurements in the context of patient age to avoid unnecessary interventions [15]. Second, the moderate influence of weight suggests that population-specific reference ranges may need adjustment in societies with a high prevalence of obesity [16]. Third, the lack of significant effect from sex and height simplifies interpretation by reducing the number of confounding variables [14].

From a research perspective, future studies should aim to incorporate larger and more diverse populations to validate these trends. Imaging modalities such as magnetic resonance cholangiopancreatography (MRCP) or high-resolution ultrasound may allow more precise measurement and could also help explore the role of comorbidities like gallstones, pancreatitis, or metabolic syndrome. Longitudinal studies would further clarify whether increases in the CBD size are purely age-related or partly driven by cumulative exposure to subclinical pathology.

Limitations

This study has several limitations. Since it was conducted in a single center, the findings may not be fully generalizable. The modest sample size, though age-stratified, limits external validation. Ultrasonography, despite being performed by an expert, is operator-dependent and may introduce minor bias. Only basic demographic and anthropometric variables were assessed, while other potential factors such as metabolic profile, and comorbidities were not included.

## Conclusions

This study demonstrated that the diameter of the CBD increases significantly with age, making age the most influential predictor among the variables assessed. Body weight shows a moderate positive association with CBD size, while height has a weak influence, and sex is not a significant determinant. These findings support the use of age-adjusted reference values for interpreting CBD measurements in clinical settings. While weight may further inform diagnostic considerations, height and sex appear to hold limited relevance. Future research involving diverse populations and longitudinal designs is essential to refine normative standards and improve diagnostic accuracy in biliary imaging.
